# Providing comprehensive health services for young key populations: needs, barriers and gaps

**DOI:** 10.7448/IAS.18.2.19833

**Published:** 2015-02-26

**Authors:** Sinead Delany-Moretlwe, Frances M Cowan, Joanna Busza, Carolyn Bolton-Moore, Karen Kelley, Lee Fairlie

**Affiliations:** 1 Wits RHI, Faculty of Health Sciences, University of the Witwatersrand, Johannesburg, South Africa; 2 CeSHHAR Zimbabwe, Harare, Zimbabwe;; 3 Department of Infection and Population Health, University College London, London, United Kingdom; 4 Department of Population Health, London School of Hygiene and Tropical Medicine, London, United Kingdom; 5 Centre for Infectious Disease Research in Zambia (CIDRZ), Lusaka, Zambia

**Keywords:** adolescent, youth, injecting drug use, MSM, sex workers, risk, integrated services

## Abstract

**Introduction:**

Adolescence is a time of physical, emotional and social transitions that have implications for health. In addition to being at high risk for HIV, young key populations (YKP) may experience other health problems attributable to high-risk behaviour or their developmental stage, or a combination of both.

**Discussion:**

We reviewed the needs, barriers and gaps for other non-HIV health services for YKP. We searched PubMed and Google Scholar for articles that provided specific age-related data on sexual and reproductive health; mental health; violence; and substance use problems for adolescent, youth or young sex workers, men who have sex with men, transgender people, and people who inject drugs.

**Results:**

YKP experience more unprotected sex, sexually transmitted infections including HIV, unintended pregnancy, violence, mental health disorders and substance use compared to older members of key populations and youth among the general population. YKP experience significant barriers to accessing care; coverage of services is low, largely because of stigma and discrimination experienced at both the health system and policy levels.

**Discussion:**

YKP require comprehensive, integrated services that respond to their specific developmental needs, including health, educational and social services within the context of a human rights-based approach. The recent WHO *Consolidated Guidelines on HIV Prevention, Diagnosis, Treatment and Care for Key Populations* are an important first step for a more comprehensive approach to HIV programming for YKP, but there are limited data on the effective delivery of combined interventions for YKP. Significant investments in research and implementation will be required to ensure adequate provision and coverage of services for YKP. In addition, greater commitments to harm reduction and rights-based approaches are needed to address structural barriers to access to care.

## Introduction

Young people aged 10–24 represent at least a quarter of the world's population, but are disproportionately affected by HIV [1,2]. Globally, the health of young people is important as it is an indicator of future population health, and also social and economic development. Although the rate of new HIV infections has declined or stabilized in many populations, over a third of new HIV infections continue to occur among the 15- to 24-year age group [3]. HIV risk and prevalence are not uniform; people who sell sex (SW), inject drugs (PWID), men who have sex with men (MSM) and transgender people (TG) have been shown to have higher risks for HIV infection than the general population [4–6]. Many practice more than one risk behaviour. For example, young MSM may also use drugs and sell sex for drugs, emphasizing the need for comprehensive, integrated health services. Key populations (KP) contribute disproportionately to HIV transmission dynamics within countries, with recent estimates suggesting that 50% of new HIV infection occur among these populations [7]. In addition to their high-risk behaviour, KP frequently experience significant stigma, discrimination and violence, which further limits their ability to adopt preventive behaviours and access health services [8]. In YKP, the effects of stigma, discrimination and violence are exacerbated by policy and legal barriers related to the age of consent for sex as well as selected medical interventions, further limiting access to a range of health services [7]. As a consequence, YKP are frequently a hidden population, and reliable and representative epidemiological data on their health are scarce [7]. This paucity of data often leads to neglect of their specific needs by programmes designed either for young people more generally, or for adult KP. Failure to identify the comprehensive health needs of YKP, and their specific barriers to care, has the potential to undermine the success of HIV prevention programmes targeted at these populations [9].

### Conceptual framework for adolescent health

**Figure 1:**
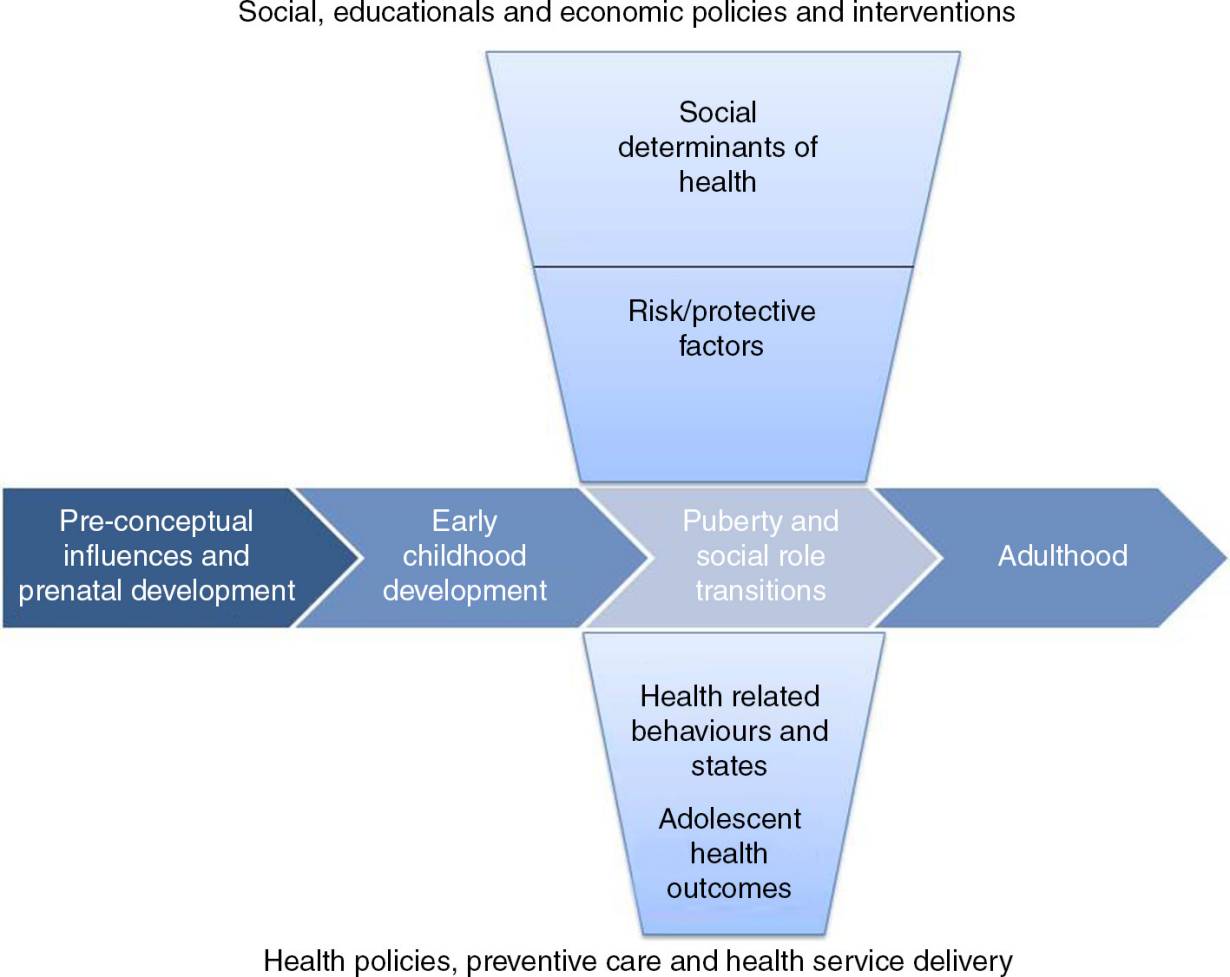
Conceptual framework of adolescent health, adapted from Sawyer et al. [9].

While YKP require specific interventions for the prevention, treatment and care of HIV, YKP also require non-HIV-related health services that respond to the health needs of their particular developmental life stage. The complex physical, psychological, emotional and social changes that take place during adolescence have immediate and long-term implications for individuals [9]. For example, the onset of puberty is linked to the initiation of sexual activity, and subsequent exposure to the risk of pregnancy and STIs, including HIV. Awareness of sexual orientation emerges during this period. Mental health disorders also emerge during the second decade of life. High rates of self-harm are observed in young people, and suicide is a leading cause of death [10]. Increased risk-taking and a heightened sensitivity to peers may influence adolescent experimentation with substance use. Although risk-taking is considered a normal part of adolescent development, risk-taking by YKP can have serious adverse consequences. More than any other life stage, adolescent health is strongly determined by social context. Both structural determinants of health (e.g. national wealth, income inequality, access to education and health services, employment opportunities and gender inequality) and more proximate determinants of health (e.g. connectedness of adolescents to family and school) affect health-related behaviour and outcomes during adolescence [11]. It is not surprising therefore that poor sexual and reproductive health (SRH), mental health disorders, violence and injury, and substance use account for the majority of disability and disease experienced by people aged 10–24 globally [12]. Sawyer and colleagues have proposed a conceptual framework to enhance our understanding of adolescent health and development (Figure 1) [9]. The horizontal axis describes a life-course perspective from the pre-conceptual and prenatal period through to adulthood. The vertical axis describes the social determinants of health and the pathways by which these influence health outcomes. The nexus of these two axes is the period of adolescence, a time of enormous physical, emotional, mental and social transition. Policy and programmatic responses to adolescent health operate along the vertical axis, but should not be developed without an appreciation for the importance of adolescence within a life-course perspective [9]. Guided by this conceptual approach, we review the needs, barriers and gaps for non-HIV-related services for YKP as part of a special series of papers on YKP.

## Methods

We focused our review on those non-HIV-related services that are developmentally relevant to this population based on the conceptual framework outlined above, and/or included as recommended interventions in the recently published WHO *Consolidated Guidelines on HIV Prevention, Diagnosis, Treatment and Care for Key Populations* (Table 1). We undertook a targeted, web-based search to identify age-specific data on the health needs and barriers to care for YKP aged 10–24. Using PubMed and Google Scholar, we focused on articles published in English since 1990, with a particular emphasis on systematic reviews and more recent publications. In order to identify age-specific data or references we used the key words “young,” “youth,” “adolescent,” and “age” in combination with search terms for each KP (e.g. “MSM,” “men who have sex with men,” “gay,” “bisexual”) and for each health topic (e.g. “condoms,” “unprotected sex,” “STI,” etc.). Abstracts were retrieved and read, and if relevant age-related data were provided, full-text articles were retrieved. Over a 2000 abstracts were identified in the initial searches, but far fewer contained age-specific information relevant to YKP and 110 articles with age-specific information were included. Due to the paucity of age-specific data on YKP, articles that referred to KP or adolescent populations were used to supplement searches.

**Table 1:** Summary of relevant WHO recommendations for linked services key populations [7]

**HIV Prevention**
**Post-exposure prophylaxis (PEP)** should be available to all eligible people from key populations on a voluntary basis after possible exposure to HIV.
All pregnant women from key populations should have the same access to services for **prevention of mother-to-child transmission (PMTCT)** and follow the same recommendations as women in other populations.
**Harm reduction**
All people from key populations with **harmful alcohol or other substance use** should have access to evidence-based interventions, including brief psychosocial interventions involving assessment, specific feedback and advice.
**Prevention and management of co-infections and co-morbidities**
Key populations should have the same access to **hepatitis B and C** prevention, screening and treatment services as other populations at risk of or living with HIV.
Routine screening and management of **mental health disorders** (depression and psychosocial stress) should be provided for people from key populations living with HIV to optimize health outcomes and improve their adherence to ART. Management can range from counselling for HIV and depression to appropriate medical therapies.
**Sexual and reproductive health**
Screening, diagnosis and treatment of **sexually transmitted infections** should be offered routinely as part of comprehensive HIV prevention and care for key populations.
People from key populations, including those living with HIV, should be able to experience full, pleasurable sex lives and have **access to a range of reproductive options**.
**Abortion laws and services** should protect the health and human rights of all women, including those from key populations.
It is important to offer **cervical cancer screening** to all women from key populations.
(Note: for adolescent populations, HPV vaccination is an additional recommendation for prevention of HPV-associated disease including anogenital cancers).
It is important that all women from key populations have the same support and access to services related to **conception and pregnancy care**, as women from other groups.
**Critical enablers**
**Laws, policies and practices** should be reviewed and, where necessary, revised by policymakers and government leaders, with meaningful engagement of stakeholders from key population groups, to allow and support the implementation and scale-up of health care services for key populations.
Countries should work towards implementing and enforcing **anti-discrimination** and protective laws, derived from human rights standards, to eliminate stigma, discrimination and violence against people from key populations.
Health services should be made **available, accessible and acceptable** to key populations, based on the principles of medical ethics, avoidance of stigma, non-discrimination and the right to health.
Programmes should work towards implementing a package of interventions to enhance **community empowerment** among key populations.
**Violence** against people from key populations should be prevented and addressed in partnership with key population-led organizations. All violence against people from key populations should be monitored and reported, and redressal mechanisms should be established to provide justice.

## Results

Poor SRH outcomes, mental health disorders, violence and injury, and substance use account for the majority of disability and disease experienced by young people aged 10–24 globally [12]. We summarized the findings of our review which demonstrate overall that YKP experience an even higher burden of disease than older KP, as well as a higher burden of disease than their age peers in the general population.

### Unprotected sex

Unprotected sex is an important risk factor for negative health outcomes among young people, accounting for 4% of disability-adjusted life years (DALYs) in those aged 10–24 [12]. Several studies have shown younger age is associated with more frequent unprotected sex among KP [13], often due to lower levels of education, knowledge, and risk perception [13–15]. Young key populations (YKP) may also be unaware of where to access condoms and other contraception. The combination of low condom self-efficacy and more frequent sex or change in sexual partners also puts YKP at higher risk of sexually transmitted infection (STI) [16–18]. For many, the ability to negotiate safer sex with partners is limited by imbalances in relationship power, compounded by adolescent aspirations for love and intimacy. For example, although condom use rates may be higher among sex workers than the general population, several studies have shown how younger sex workers may be less experienced than older sex workers with condom negotiation, and more vulnerable to being forced to have sex without a condom either by clients or managers [19,20]. Unprotected sex may also be associated with expressions of intimacy or trust, with higher rates for sex with primary partners, who are often older and thus have had a longer period of potential exposure to HIV and other STI [21–23]. In one study of young MSM aged 16–20, considering a relationship to be serious was associated with an eight-fold increase in the rate of unprotected sex [24]. For TG women, condom negotiation may be more difficult given their female gender identity and socially constructed role, and young TG people may be more likely to have unprotected sex to validate their gender identity [13]. Among young PWID, low rates of condom use are often associated with other high-risk practices such as needle sharing or smoking drugs together [23]. Among young PWID, there are gender differences in risk with higher rates of unprotected sex and sexual risk observed in young women [25]. Given the lower levels of condom use among YKP, the potential for negative health outcomes in this population is high.

### Sexually transmitted infections

The prevalence of STIs is also higher in YKP than among older KP peers. Studies show elevated rates of syphilis [26], gonorrhoea [27], chlamydia [28,29] and herpes simplex [30] among YKP compared with adult KP. Young MSM and TG may be more likely to have anal or rectal infections that are asymptomatic and/or remain undiagnosed [31]. STIs are also more common in those populations with more than one risk behaviour. For example, a prospective study among PWID in British Columbia, Canada, found that incident STIs were more frequent among those involved in the sex trade compared with those who did not sell sex, over a three-year period [32]. In addition to causing significant morbidity and mortality, STIs also increase the risk of HIV transmission.

Two of the viral STIs associated with cancer outcomes are now vaccine preventable. HPV is a common STI that causes cervical and other anogenital cancers. A high prevalence of HPV infection has been observed in adult KP. In a study of anal HPV prevalence and risk factors among men in Brazil, Mexico and the United States, among MSM, younger age was associated with increased prevalence of any anal canal HPV [33]. YKP may be exposed to HPV earlier and may be more at risk of developing of pre-neoplastic and neoplastic lesions in later life, especially if they are co-infected with HIV. Although access to HPV vaccination has expanded significantly in the past five years, young MSM or TG women may not benefit from vaccination programmes targeted at young girls, and may not receive the benefit afforded to heterosexual men through herd immunity [34].

YKP may be at increased risk for viral hepatitis. Hepatitis C virus (HCV) incidence in young PWID is high, raising concerns about the prevention and control of an expanding epidemic in young people [35]. A study on young PWID in Afghanistan showed that risk of HCV infection increased with each additional year of injecting among young PWID [36]. Young female PWID were reported to have a higher incidence of HCV, associated with higher risk injection practices, when compared to young men [37]. Young MSM are also at increased risk of viral hepatitis A and B [38]. YKP would benefit from vaccination against hepatitis A and B in settings where universal childhood vaccination is not routine. Where vaccination is offered to MSM, coverage is still relatively low, although promising data from several studies show that vaccine uptake is associated with younger age [39].

### Reproductive health

In addition to STIs, a frequent outcome of unprotected sex in female YKP is unintended pregnancy, which during adolescence can pose particular risks to both mother and infant [40]. Data on pregnancy intentions, outcomes, and use of contraception or prevention of mother-to-child-transmission (PMTCT) in YKP are limited. Studies in adult female sex workers show that pregnancy is frequently unwanted, termination of pregnancy is common and contraceptive use, including of emergency contraception, is low, suggesting significant unmet needs [41–43]. A recent study among Chinese adolescent sex workers showed that a quarter had never used a modern contraceptive method [44]. The main method of pregnancy prevention in this population was condoms, although condom use was often inconsistent. Half of those interviewed reported a previous induced abortion, although only a third of those had sought care from public sector services. These data are supported by another study in Chinese sex workers showing that younger sex workers were less likely to terminate their pregnancy, but those that did were more likely to seek termination from informal providers [45]. For HIV-positive YKP, PMTCT is a priority. While few data are available on access to PMTCT in YKP, given high rates of HIV infection in this population, there is likely to be a significant need. In a study of PWID in Ukraine, PWID were more likely than non-PWID to be diagnosed during labour and to have more advanced HIV disease, but less likely to receive prophylaxis or HAART to prevent vertical transmission. As a consequence, vertical transmission rates in this population were higher than in the general population [46].

Young TG have specific health needs related to their gender identity. Hormone therapy may have significant benefits for TG people, but access is frequently limited by cost or provider attitudes. As a consequence, some TG may seek hormone therapy from the non-medical sources [47], despite potential side effects from unmonitored treatment including overdose [48], or the risk associated with injecting hormones or silicone [49]. In some cases, young TG may engage in sex work to fund treatments [50]. Services for YKP need to be able to provide reliable, evidence-informed information regarding TG-specific medical and surgical procedures.

### Sexual assault

YKP are more likely to require sexual assault services including access to post-exposure prophylaxis (PEP). While no age-specific data were provided, a recent systematic review of the prevalence and correlates of violence against sex workers estimated that the lifetime prevalence of any violence ranged from 45% to 75% [51]. Young trafficked sex workers may have experienced violent rape in order to coerce them to sell sex [18,52]. Young male sex workers are not immune, and studies show that they also experience verbal, physical and sexual abuse [53]. In a study of MSM in Thailand, 18% had experienced forced sex, the majority by someone they knew, and the forced sex occurred more than once, with the first experience occurring during adolescence [54]. While estimates of the prevalence of sexual assault among PWID are limited, a recent study among women aged 16–29 attending family planning services in Pennsylvania, USA, showed 11% of women had experienced sexual violence in the previous three months, and that sexual assault was associated with injection drug use, their own or their partners [55]. Experiences of violence are strongly associated with increased risk behaviour and risk for HIV [18,56,57], as well as other negative SRH outcomes [58,59]. Interventions that address sexual assault and that provide access to PEP are a critical component of a package of services for YKP [7].

### Mental health

While emergence of mental health problems in the second decade of life is common, data suggest that YKP experience higher rates of mental health problems when compared with their same age counterparts in the general population, or older key population peers. For example, a study of young SW in China showed that those younger than age 20 experienced the highest rates of depression, suicide and substance use compared to those older than 20 [60]. A study in lesbian, gay, bisexual, and transgender (LGBT) youth in the US observed higher rates of mental disorders in this population compared to the general population [61]. Major depression, personality and substance use disorders are more common in young PWID [62], with higher rates of mental health disorders observed in young women [63].

Among the factors that influence poor mental health in young KP, stigma, discrimination, social exclusion and victimization are substantial contributors. For example, a study among young PWID in Russia showed that a large proportion had experienced discrimination resulting in loss of jobs, lack of access to health care and being forced from their family homes. Over one-third had clinical depression [64]. A study in Chinese sex workers showed that the majority had high levels of self-stigma, and that this was significantly associated with poor mental health [65]. In a US study of young MSM, experiences of victimization were strongly associated with a syndemic of depression, substance use, risky sex and intimate partner violence; these factors were also strongly associated with an increase in suicide attempts [66]. These findings were echoed in a global study of MSM in 151 countries [67], and a study in young TG women [68]. Violence experienced at the hands of family, partners, clients or police is associated with increased reporting of poor mental health outcomes [69–72].

### Substance use

Substance use and experimentation are common in adolescence, but evidence suggests that YKP are more likely to initiate substance use at an earlier age, to engage in polysubstance use and to experience more rapid increases in substance use over time [73,74]. This is of particular concern given the findings that substance use in adolescence may be more harmful to brain function and behaviour while the brain is still developing [75]. YKP may frequent social spaces where alcohol and drug use are tolerated and consumption normalized. YKP may initiate substance use to self-medicate against the anxiety associated with group behaviours, or associated negative experiences [76,77]. A study in Australian LGBT youth highlighted a higher prevalence of alcohol and drug use in this population compared to the general population. In this study, alcohol use was significantly higher in those younger than 18, and those who believed that homophobia influenced alcohol and drug use were significantly more likely to use alcohol or drugs [78]. Substance use may increase sexual desire, lower inhibitions and impair decision-making. A study of young TG women in the US documented that this group was significantly less likely to use condoms with main partners while under the influence of drugs or alcohol. Substance use may reduce an individual's concerns about safe sex in the face of other, more important desires and immediate priorities [22]. Several other studies among YKP have shown that drinking beforehand can be associated with unprotected sex and injecting behaviours [76,79,80].

The risks associated with injecting drug use are disproportionately high. Problem drug use often starts with recreational drug use, and studies show that the transition from non-injecting to injecting drug use in young people is rapid and high [77]. Early initiation of injecting drug use is associated with younger age and being female [25,81]. Young PWID are frequently initiated into drug use by peers [82,83]; young women more often are initiated by a sex partner [77,84]. A study in Ukraine reported that in 56% of boys and 72% of girls, the first injection was unplanned and often occurred after exposure to injecting among friends, with around 32% of girls initiated by their sexual partners [85]. Young PWID are more likely to inject in groups, and develop rituals associated with injecting that expose them to sharing of non-sterile equipment. Studies in young PWID show more frequent sharing of equipment, more frequent injecting and injecting in public spaces [86,87]. Young PWID are more likely to practice unprotected sex, have increased numbers of partners, or trade sex for drugs than their older peers [88,89]. Young PWID may also be less likely to engage in care for their addiction, and are also more likely to report relapse after treatment [72,90]. Young PWID have much higher mortality rates than their peers in the general population, associated with overdose or injury [91]. They have a substantial need for harm reduction and addiction treatment services, as well as links to services to address their integrated health and social needs.

### Educational, vocational and social support

In addition to health care, YKP frequently require social support because of their life stage as well as socio-structural factors that influence their behaviour. YKP may be orphaned or rejected by their families, experiencing homelessness, food insecurity and economic instability [56,92,93], and may often prioritize food, shelter and money over health [94]. Female YKP may have concerns about the welfare of their own children [95]. Access to social support and benefits is therefore essential to reducing their risk. Completion of education, initiating employment and transitioning out of the childhood home may be considered a normal part of adolescent development, yet YKP may not experience these positively. For example, school bullying and victimization may severely impair educational attainment and future employment opportunities of young MSM and TG [96]. For some, low educational attainment may become a reason to sell sex [97]. Young PWID who drop out of school may be at higher risk for HIV [15]. Linkage to educational and vocational support interventions are an important part of a developmentally appropriate response to YKP, and while not directly linked to HIV programme activities educational and vocational interventions may be critical enablers for HIV programme success in this age group.

### Barriers to care

Adolescence is marked by high rates of attrition along the continuum of HIV prevention, diagnosis and treatment services. YKP are less likely to be engaged in care [98], and coverage of services is low. While data are limited for YKP, evidence from young adult and adult populations shows that KP experience poor access to condoms and HIV testing [99], may present later for HIV treatment [100] and have lower rates of adherence [101], viral suppression [102] and retention in care [103]. PMTCT outcomes and access to linked mental health, substance use and SRH services are poor [46,104]. PWID may experience difficulty in accessing safe injecting equipment or treatment for dependence. Using the conceptual framework (Figure 1), reasons for poor access to care can be categorized as individual-level, health-system-level or structural-level barriers, and are common to all YKP.

### Individual-level barriers to care

Low levels of education and HIV knowledge or risk perception are associated with low uptake of HIV services [13]. YKP with less formal education and/or less sex education may be less familiar with what constitute safe sex or safe injection practices. YKP with internalized stigma experience more social isolation and are less able to ask trusted adults for support in decision-making [105]. They may also experience bullying by older KP [106]. YKP who have experienced poor mental health, violence or low levels of social support may have lower levels of self-efficacy for health-seeking [107].

### Health-system-level barriers to care

Perhaps the most significant barrier to health-seeking among YKP is the experience of stigma, discrimination or victimization at the hands of health care providers (HCP). In a study of young migrant sex workers in North Vietnam, despite health care being available, the young women perceived the stigma attached to sex work as a barrier to receiving health care, and preferred to receive health education and care from peers [52]. In another study involving male, female and TG sex workers in Africa, denial of treatment for injuries following physical assault or rape and general hostility from public sector providers were common [108]. Similar experiences were reported by PWID in India [70]. Younger PWID expressed a preference for syringe-dispensing machines over staffed needle exchange programmes because of their desire to hide their identity or because they did not like the way they were treated at staffed services [109]. Concerns about privacy and confidentiality are an important barrier to care. In a US-based study, LGBT youth expressed greater concerns about confidentiality and were less likely to seek care from school-based services compared to heterosexual peers [110]. In addition to concerns about poor attitudes, HCP may not have sufficient skill, competence or training to deal with the specific health and social needs of YKP. A US survey of HCP reported that the majority of respondents would not regularly discuss sexual orientation, sexual attraction or gender identity while taking a sexual history from a sexually active adolescent. The majority of physicians did not believe that they had all the necessary skills to address issues of sexual orientation with adolescents [111]. Studies reveal that provider willingness to answer questions, their respect for and understanding of adolescents and the responsiveness of the social and physical environment towards youth are all associated with young people's intention to seek and engage in care [112]. Negative experiences with providers may prompt YKP to seek care from non-conventional services [47]. For YKP, cost and waiting time are also barriers to care [103]. Young people are less likely to have access to ready cash and may have competing demands or less control over their time. In several studies, YKP highlight the importance of integrated services that address their multiple health needs [113]. Lack of service integration adds time and cost to clinic visits and may be a further barrier to care. In many cases, services for KP may not be sufficiently “youth-friendly.” Providers may not have an appreciation for the specific health and communication needs of YKP. YKP may experience discomfort when seeking care with adults. Location and transport may also be a barrier to care. Larger, more formal venues can enhance prevention initiatives, including on-site services. Geographical targeting of services for YKP can be complicated by the social and sexual networking patterns of YKP who may find partners through the Internet, meet them in informal venues, and generally be more mobile. This can increase the risk involved and makes it harder for services to identify and reach them [114].

### Structural-level barriers

Criminalization reduces YKP's control over their behaviour, impedes their access to health services and obstructs health-service provision and legal protection. In many settings, YKP are criminalized for their behaviour/s and risk incarceration [115,116]. Even in settings where activities are not criminalized, they may experience significant stigma, discrimination or police harassment, as a result of both their group identity and their age [117–119]. Studies of MSM in African countries where homosexuality is criminalized demonstrate how criminalization makes MSM more vulnerable to violence and less able to access health care or preventive services [99,119,120]. YKP are vulnerable to harassment and exploitation by the police, and may go to substantial lengths to avoid police. A mapping study in Canada showed a significant geographic relationship between a heavily concentrated core area of health and syringe availability and avoidance of these settings by substance using street-based sex workers due to policing; this correlation was strongest among younger women [121]. Several studies have highlighted how police arrest YKP for carrying drug paraphernalia or confiscate it without arrest [70,122]. Arrest and detention are frequently associated with police beatings; a Thai study among young PWID showed that younger age was associated with more frequent reports of police beating [118]. In addition to physical violence, YKP are also vulnerable to sexual coercion and violence at the hands of authorities [123].

Access to care is further confounded by the legal status of YKP as minors. In many countries, adolescents require parental permission to access testing, treatments or procedures. This is a particular problem for minors who do not live with their parents or do not wish to disclose their behaviour to them. For example, young women seeking contraception or safe abortion services are likely to seek care outside of conventional health services [45]. In many countries where opioid substitution services are available, age restrictions are placed on them [124]. In some settings, HCP are legally obliged to report underage sex or other illegal activities. This may compromise disclosure and provision of adequate care for risky behaviours. Age restrictions are also placed on access to housing and other social services which may be needed.

An absence of clear legal status may also be a barrier for access to health services. TG people may experience significant barriers to obtaining services, legal entitlements and legal protection because the gender assignment on their administrative documents may be in conflict with their gender identity [113]. Migrant YKP may also not have adequate legal documentation of citizenship, and may not be able to access services in their host country. As adolescents, they are often dependent on parents for the provision of information necessary for citizenship or travel documentation. The absence of appropriate official documentation may make them vulnerable by limiting their access to health and social services and benefits that they might otherwise be entitled to.

Many YKP experience additional stigma and discrimination associated with their racial or ethnic identity, in addition to their group identity. There is overwhelming evidence from the United States that young Black MSM have the highest concentration of HIV of any sub-population despite little evidence of higher risk behaviour. Instead, social and structural factors act as barriers to health care access [125]. In other settings, YKP from either indigenous or migrant populations are marginalized and have limited access to health services [87].

## Discussion

Despite the paucity of age-specific data for YKP, this review confirms that in addition to interventions for the prevention, treatment and care of HIV, YKP also require other, non-HIV-related health services that respond to their significant health and development needs as adolescents. The WHO recommendations for a comprehensive package of services that includes SRH services and care for mental health disorders is an important first step in recognizing the impact of these other health concerns on the success of HIV prevention, treatment and care interventions [7]. These guidelines specifically recognize the health and developmental needs of YKP, and provide commentary on specific considerations for the delivery of health sector interventions to YKP.

The next priority is to ensure that these recommendations are implemented at scale and with sufficient intensity to ensure an impact on the HIV epidemic, and the health of YKP. While there is substantial evidence for effective interventions to prevent and treat HIV infection in adults, less is known about the delivery of these interventions to adolescents [126]. Current coverage of services for YKP is generally low [127,128], and consideration of optimal service delivery models that respond to current barriers to care are now a priority. The requirement to make services accessible, acceptable and available to YKP provides an opportunity to evaluate interventions aimed at addressing health system barriers to care. Beyer and colleagues have proposed three models of service provision for KP: integrated models of care, stand-alone models of care or hybrid models of service provision [8]. Integrating HIV and related service provision for YKP into primary health care (PHC) offers significant potential for expanding coverage and access to care for YKP, and may be the only option for service delivery in some settings. Integrated models have the potential to address several of the health systems barriers. Sensitization of services and training of all staff in facilities, not just HCP, is a potentially powerful structural intervention to enhance the effectiveness of HIV programmes for YKP and reduce stigma more generally. There are a number of positive approaches to stigma reduction, with growing experience on how to work with HCP and communities to reduce anticipated and enacted stigma [129]. There is accumulating evidence to suggest that interventions using a combination of sensitization and participatory activities can reduce HIV stigma in health care [130,131] and community settings [132,133]. A recent systematic review identified 48 evaluations in which HIV-related stigma was assessed as an outcome [134]. While the studies found that information, skills building, counselling and PLHIV testimonials were associated with less stigmatizing attitudes among participants, the evidence base had many gaps. Training and sensitization of HCP to the needs of MSM has also been shown in a study in Kenya to reduce homophobic attitudes up to three months after training [135]. In addition to stigma reduction interventions, initiatives to make PHC more “adolescent and youth-friendly” are likely to benefit the subset of YKP. Evidence from several systematic reviews confirms that implementation of a combination of interventions, including training of HCP, outreach activities and out-of-facility services tailored to context and target population, demonstrated some impact on uptake of health services by young people [126], although training of service providers in adolescent-friendly service provision alone appears to be less beneficial [128].

Peer approaches are potentially a critical component of services for YKP, given the particular developmental susceptibility of adolescents to peer influence. Peers are in a unique position to identify and reach out to YKP who may be experiencing barriers to health care through lack of knowledge, risk perception or self-efficacy. Evidence from a systematic review of interventions to improve linkage and retention in HIV care in low- and middle-income countries supports integrating formalized care with peer support to increase the uptake of HIV services, although data on adolescents are limited [136]. While peer interventions for YKP have been found to be positively associated with increased knowledge and condom use in some programmes [13], they are optimal when included as part of a comprehensive empowerment approach [137]. There is growing evidence that empowerment approaches for SW in particular improve HIV programme outcomes [138]. In addition, empowerment approaches are likely to have benefits for other health challenges, particularly violence. Evidence from programme assessments show that it is possible to prevent violence using empowerment approaches, with some interventions achieving significant effects within programme timeframes [139]. Integrated services delivered at scale offer a significant platform for the delivery of community mobilization and empowerment interventions. Despite the growing evidence that empowerment approaches produce health benefits, effective implementation of empowerment processes within many settings, particularly in Africa, has been limited [140]. Challenges to the sustainability of empowerment interventions include lack of social cohesion within transient communities, limited capacity and resources, and variable commitment of programmers to empowerment interventions. Given these challenges, and the fact that integrated models may not sufficiently address the barriers presented by lack of privacy and accessibility, cost or waiting times, or the need for access to a range of non-health services, alternative models warrant further exploration.

While there is a precedent for stand-alone models of service delivery for KP [141], they may not be suitable in many settings. While these services may be able to provide KP-sensitive services, they may also increase stigma and marginalization, and provide targets for attack. An alternative to stand-alone facilities are out-of-facility-based delivery strategies. Currently data are limited on the benefits of out-of-facility-based approaches to health care delivery among adolescents, although two reviews suggest that services delivered through mixed-use youth centres are not well-used or particularly effective for adolescents in the general population [128]. The authors do note, however, the absence of studies or evaluations examining outcomes among vulnerable or marginalized adolescents. Programmatic experience suggests that drop-in centres provide a valuable opportunity to offer a range of services specific to the needs of YKP. For example, as an alternative to integrating adolescent PWID into programming targeted at adult injectors, who can appear threatening and model harmful behaviours, Moldovan NGOs established drop-in centres welcoming adolescents with overlapping risks, including those living on the street, injecting drugs or involved in sex work. A case management approach then linked individuals to a network of health and social services [142]. Linkage to non-health services is generally valued by YKP [103,113], and may even encourage retention in care. Medical and food incentives have been found to increase retention in care prior to antiretroviral treatment initiation [136], emphasizing the importance of non-HIV-related service delivery provision. Other out-of-facility options include the training of pharmacist to counsel and provide adolescents with appropriate needle exchange and drug substitution services [143]. Work place policies have been used with particularly good effect in sex work establishments in Asia; however, there is less evidence for how these policies may benefit YKP [13]. In these cases, strong linkages with child protection services ensure enforcement of anti-trafficking laws. While schools are an important venue for the delivery of health education, they may not be an optimal for the delivery of services to YKP and should be used to complement, not replace, health care services for adolescents located outside schools [126].

Internet-based interventions represent a different type of out-of-facility service. The rapid expansion of access to the Internet and social media in the past two decades, even in low- and middle-income countries, through mobile phone technology, represents a significant opportunity to engage with previously hidden populations, or those that are socially or geographically isolated [144,145]. The Internet provides a novel way to expand access to standardized information, to build virtual communities of supportive peers and to link YKP to services. There is accumulating evidence of the acceptability of delivering digital-based media interventions to adolescents, KP or YKP in settings in North America [146–148], South America [149], Asia [150,151] and Africa [152], although there is less evidence on the impact of these services on longer term health outcomes [153]. A recent review evaluated the impact of digital-media-based interventions on sexual health knowledge, attitudes and/or behaviours of adolescents in the general population aged 13–24 [154]. Of the ten studies reviewed, six studies increased knowledge of HIV, STI or pregnancy. A recent study among young MSM showed that an Internet-based, peer-led social media HIV prevention intervention can increase community cohesion and uptake of HIV services [155]. These and other evaluations of Internet interventions for YKP show positive short-term outcomes for health. Given initial findings from this and other similar programmes, further evaluation is needed to gauge the potential benefit of these programmes on health outcomes over a longer period [156].

Hybrid models that combine the reach of services integrated at PHC level with the peer-based, outreach and empowerment approaches offered by more flexible community-based NGOs are probably the optimal model of service delivery. Ultimately, decisions about service delivery models need to be informed by user preferences, and they need to take into account considerations that are context-specific and address the age-based needs of YKP, as well as respond to their specific risk behaviours, the epidemic setting and the social, legal and political complexities associated with service delivery for this group. To this end, efforts should be focused on making YKP more visible through research and monitoring, so that their needs are recognized and prioritized by public health systems. While the provision of linked non-HIV-specific services requires significant investment and innovation, significant gains in coverage can be achieved with modest increases in resources [157]. Finally, given the strong influence of socio-structural factors on adolescent health, coupled with the fact that many factors that influence YKP's risk are outside of their immediate control, interventions that address the structural barriers to care are a critical part of an effective HIV response for YKP. In addition to changes in laws and policies that promote stigma and discrimination, specific interventions that address the age of consent are more essential for this age group.

## Conclusions

Despite the dearth of age-specific data, YKP have significant non-HIV-related health needs, and face significant obstacles to accessing care as a result of their age and membership of KP. While YKP face significant hardship and risk, they also represent the greatest hope for reducing the harms associated with their behaviours, and preventing new HIV infections. Now that normative guidance exists for the optimal set of interventions for KP, priority needs to be placed on evaluating optimal approaches for the delivery of a comprehensive package of care of YKP. Investments in providing linked, non-HIV but related services that also address critical enablers of programmes are likely to have significant benefits for HIV prevention across all populations.
